# Development of CRISPR Interference (CRISPRi) Platform for Metabolic Engineering of *Leuconostoc citreum* and Its Application for Engineering Riboflavin Biosynthesis

**DOI:** 10.3390/ijms21165614

**Published:** 2020-08-05

**Authors:** Jaewoo Son, Seung Hoon Jang, Ji Won Cha, Ki Jun Jeong

**Affiliations:** 1 Department of Chemical and Biomolecular Engineering, BK21 Plus program, KAIST, 291 Daehak-ro, Yuseong-gu, Daejeon 34141, Korea; jwson90@kaist.ac.kr (J.S.); s.h.jang0314@gmail.com (S.H.J.); jiwoncha@kaist.ac.kr (J.W.C.); 2 Institute for The BioCentury, KAIST, 291 Daehak-ro, Yuseong-gu, Daejeon 34141, Korea

**Keywords:** lactic acid bacteria, *Leuconostoc citreum*, CRISPRi, synthetic biology, riboflavin

## Abstract

*Leuconostoccitreum*, a hetero-fermentative type of lactic acid bacteria, is a crucial probiotic candidate because of its ability to promote human health. However, inefficient gene manipulation tools limit its utilization in bioindustries. We report, for the first time, the development of a CRISPR (Clustered Regularly Interspaced Short Palindromic Repeats) interference (CRISPRi) system for engineering *L. citreum*. For reliable expression, the expression system of synthetic single guide RNA (sgRNA) and the deactivated Cas9 of *Streptococcus pyogenes* (SpdCas9) were constructed in a bicistronic design (BCD) platform using a high-copy-number plasmid. The expression of SpdCas9 and sgRNA was optimized by examining the combination of two synthetic promoters and Shine–Dalgarno sequences; the strong expression of sgRNA and the weak expression of SpdCas9 exhibited the most significant downregulation (20-fold decrease) of the target gene (sfGFP), without cell growth retardation caused by SpdCas9 overexpression. The feasibility of the optimized CRISPRi system was demonstrated by modulating the biosynthesis of riboflavin. Using the CRISPRi system, the expression of *ribF* and *folE* genes was downregulated (3.3-fold and 5.6-fold decreases, respectively), thereby improving riboflavin production. In addition, the co-expression of the *rib* operon was introduced and the production of riboflavin was further increased up to 1.7 mg/L, which was 1.53 times higher than that of the wild-type strain.

## 1. Introduction

*Leuconostoc* spp. is a hetero-fermentative type of lactic acid bacteria that plays a significant role in the fermentation of products such as kimchi, milk, vegetables and meat [1]. Recently, *Leuconostoc* spp. has been in the spotlight as one of the crucial candidates for use as a probiotic, as it has the ability to produce vitamins and bioactive antimicrobial peptides, and has also been shown to promote human health by regulating immune responses [2,3,4]. Furthermore, it has distinct advantages, such as its categorization as a “Generally Recognized As Safe” (GRAS) strain [5], its enhanced protein secretion ability [6,7], and its capacity to produce exopolysaccharides that are recognized to have therapeutic properties [8,9,10]; thus, *Leuconostoc* spp. is desirable as a next-generation probiotic that can be used to deliver pharmaceutical proteins in humans or animals and can be applied in various biomedical fields.

**Figure 1:**
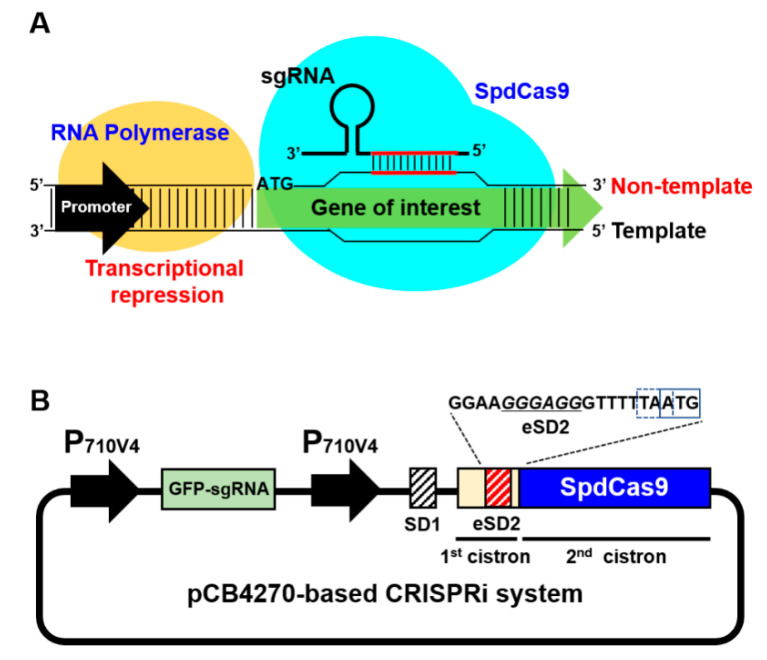
(**A**) Schematic of the CRISPRi system. (**B**) Schematic diagram of the plasmid pGFP–sgR–D1–Em constructed for the CRISPRi system for *L. citreum.* Dashed and solid boxes in the DNA sequences indicate the stop codon of the first cistron and start codon of second cistron (SpdCas9), respectively.

Although *Leuconostoc* spp. has potential advantages in a variety of bioindustry fields, extensive research has not been conducted on the engineering of *Leuconostoc* spp. due to the lack of genetic manipulation tools. Particularly, the engineering of bacterial hosts, including *Leuconostoc* spp., can enhance their performance by giving them desired properties, which can be achieved by the extensive manipulation of the host genome, such as the elimination or downregulation of unnecessary genes and/or the overexpression of essential genes. However, such manipulation of genes requires very precise tools for genome editing [11,12]. Recently, various genome-editing tools that can be used in lactic acid bacteria (LAB) have been introduced, including homologous recombination and the recombinase-mediated insertion or deletion of target genes [13,14,15]. The development of the CRISPR (Clustered Regularly Interspaced Short Palindromic Repeats)–Cas9 system as a counter-selection tool in combination with existing recombineering tools has improved the genome editing efficiency in LAB [16]. Indirect genome modification tools using catalytically deactivated Cas9 (dCas9) can make it possible to effectively regulate the expression of target genes without permanently removing the genes from chromosomes. This system, called CRISPR interference (CRISPRi), can regulate the expression of a target gene by blocking RNA polymerase through the interaction of dCas9 with synthetic guide RNA, which has a complementary sequence to the target gene (Figure 1A) [17,18]. However, most of these tools are available only for *Lactococcus* spp. and *Lactobacillus* spp.; genome-editing tools that can be optimized and used for *Leuconostoc* spp. are very limited.

Here, we developed a CRISPRi system for regulating the knock-down of gene expression in *L. citreum* and used this tool for the engineering of *L. citreum* to increase riboflavin biosynthesis. Recently, we developed several synthetic parts (high-copy-number plasmids, and promoters and Shine–Dalgarno sequences (SD) with different strengths) for the fine tuning of gene expression in *L. citreum* [19,20]. Using these synthetic parts, we first developed CRISPRi for the reliable expression of CRISPRi components such as synthetic single guide RNA (sgRNA) and the catalytically deactivated Cas9 of *Streptococcus pyogenes* (SpdCas9) [21,22]. To reduce the toxicity caused by the overexpression of SpdCas9, the expression of SpdCas9 is optimized by combining synthetic expression parts (promoters and SDs) to produce a bicistronic design (BCD) expression platform. Finally, to demonstrate the feasibility of optimized CRISPRi on the modulation of metabolite biosynthesis, we engineered the riboflavin biosynthesis pathway in *L. citreum*. The titer of riboflavin is remarkably increased by employing a combination of two strategies: (i) regulating the knock-down of the branched folate synthesis pathway and a loss of the capability of riboflavin to convert into a flavin mononucleotide (FMN) using CRISPRi and (ii) the overexpression of the *rib* operon (*ribA*, *ribG*, *ribH*, and *ribB*).

## 2. Results and Discussion

### 2.1. Construction of CRISPRi in the BCD Platform

To implement the CRISPRi platform in *L. citreum*, we employed SpdCas9 derived from a type II CRISPR system [23,24,25]. It is generally known that a high concentration of sgRNA in cells is required for the effective operation of CRISPRi [26]. Therefore, to provide a high amount of sgRNA, we first constructed sgRNA and SpdCas9 expression cassettes in a high-copy-number plasmid (pCB4270) that is present at about 60 copies per cell in *L. citreum* [19]. For the reliable expression of SpdCas9, the expression cassette was constructed in a bicistronic design (BCD) platform, which we had previously developed to induce the reliable expression of recombinant proteins in *L. citreum* [20]. In the BCD, the first cistron consisting of the first SD sequence (SD1) and a short peptide (17 amino acids) was cloned in a downstream region of the high strength constitutive promoter (P_710V4_) (Figure 1B). An engineered second SD sequence (eSD2) that had a high translational initiation property [20] was cloned in the 3’-end of the first cistron. It served as a ribosome binding site (RBS) for the translation of SpdCas9 (second cistron) by translational coupling (Figure 1B). To evaluate the operation of the CRISPRi platform in *L. citreum*, an expression cassette for sgRNA targeting the superfolder green fluorescent protein (sfGFP) gene was constructed upstream of the SpdCas9 expression cassette in the same plasmid (Figure 1B). Previous studies have shown that targeting the non-template strand of a target gene in a type II CRISPR system shows higher transcriptional repression efficiency than targeting the template strand [21,22]. We constructed GFP-sgRNA comprising a 20-base sequence complementary to the non-template target region next to the protospacer adjacent motif (PAM) sequence, located at the most upstream position in the sfGFP coding sequence (Table S3 and Figure S1A).

**Figure 2:**
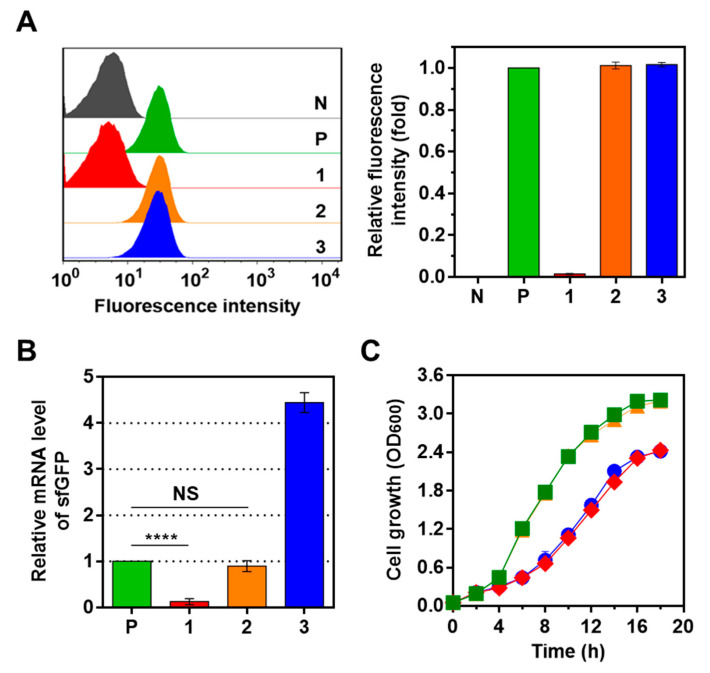
(**A**) The histogram of fluorescence-activated cell-sorting (FACS) analysis for sfGFP expression. The fold changes of fluorescence intensity in the FACS analysis were plotted in the right graph. (**B**) The quantification of sfGFP transcripts by qRT-PCR. NS represents nonsignificant (*p*-value > 0.05), **** *p*-value < 0.0001 based on Student *t*-test. N, *L. citreum* CB2567; P, *L. citreum* JW001; 1, *L. citreum* JW001 harboring pGFP–sgR–D1–Em; 2, *L. citreum* JW001 harboring pGFP–sgR–Em; 3, *L. citreum* JW001 harboring pD1–Em. (**C**) Time profile of cell growth. The growth curve was presented as an average value from the biological replicates’ cell culture. Symbols: ■; *L. citreum* JW001, ◆; *L. citreum* JW001 harboring pGFP–sgR–D1–Em, ▲; *L. citreum* JW001 harboring pGFP–sgR–Em, ●; *L. citreum* JW001 harboring pD1–Em.

The ability of the SpdCas9-mediated CRISPRi system (pGFP–sgR–D1–Em) to regulate the expression of the sfGFP gene was evaluated using *L. citreum* JW001, in which the sfGFP gene expression cassette was integrated into the *ldhD* locus of *L. citreum* CB2567. *L. citreum* JW001 harboring pGFP–sgR–D1–Em was cultured, and the change of fluorescence intensity (FI) was analyzed by a flow cytometer. As shown in Figure 2A, cells harboring pGFP–sgR–D1–Em showed a significantly reduced FI compared to that of *L. citreum* JW001 without the plasmid used as a positive control, and also showed a similar FI as that of wild-type (WT) *L. citreum* (no expression of sfGFP) as a negative control. In contrast, the cells harboring pGFP–sgR–Em (expression of sgRNA alone) or pD1–Em (expression of SpdCas9 alone) exhibited similar fluorescence intensities (FIs) as those of the positive control (*L. citreum* JW001) (Figure 2A). These results indicated that the reduced sfGFP expression level was due to the effective operation of CRISPRi, and was not a phenomenon that occurs by the expression of either sgRNA or SpdCas9.

In the CRISPRi system, an RNA–protein complex of sgRNA and SpdCas9 prevents RNA polymerase from binding to the target DNA, resulting in the repression of target gene transcription (Figure 1A) [22]. In our study, this repression, which occurred due to the cooperation of sgRNA and SpdCas9, was confirmed by analyzing the transcription level of the sfGFP gene under the co-expression of sgRNA and/or SpdCas9. After the cultivation of cells, total mRNA was purified from each strain, and the transcription level of sfGFP was quantified by quantitative reverse transcription PCR (qRT-PCR). Comparative analysis of the mRNA clearly indicated that the mRNA transcription level for the sfGFP gene was reduced by more than 90% in the cells expressing both sgRNA and SpdCas9 compared to the positive control (*L. citreum* JW001) (Figure 2B). We concluded that the constructed CRISPRi system introduced in *L. citreum* effectively regulated the expression of the chromosomal gene in *L. citreum*. In this experiment, however, one unexpected result—an abnormal elevated level of mRNA transcript for the target gene (sfGFP)—was identified in *L. citreum* JW001, harboring pD1–Em, expressing only SpdCas9 without sgRNA, while *L. citreum* JW001 harboring pGFP–sgR–Em expressing only sgRNA without SpdCas9 exhibited a similar level to the positive control (Figure 2B). In addition, when we checked the cell growth during flask cultivation, the retardation of cell growth under the overexpression of SpdCas9 (*L. citreum* JW001 harboring either pGFP–sgR–D1–Em or pD1–Em) was also observed (Figure 2C).

### 2.2. Optimization of CRISPRi System Using Synthetic Parts

Previous studies have confirmed that the overexpression of SpdCas9 in many bacteria causes cytotoxicity to host cells, which results in a significant reduction of cell growth [27]. In the case of *E. coli*, abnormal morphological changes of cells were induced, and transcriptome analysis showed that the overexpression of SpdCas9 affected the transcriptional levels of various genes in the host [28]. As shown in Figure 2, we also had a similar growth retardation problem and abnormal gene expression under the overexpression of SpdCas9. The exact mechanism of the effects of SpdCas9 overexpression on cells is not yet clearly identified; however, it is known that cytotoxicity due to the overexpression of SpdCas9 places limitations on the effective introduction of the CRISPRi system into hosts [29]. Therefore, we decided to optimize the CRISPRi system by fine tuning the expression level of SpdCas9 with synthetic parts to induce the effective operation of the CRISPRi system while reducing the cytotoxicity of SpdCas9 in *L. citreum*.

**Figure 3:**
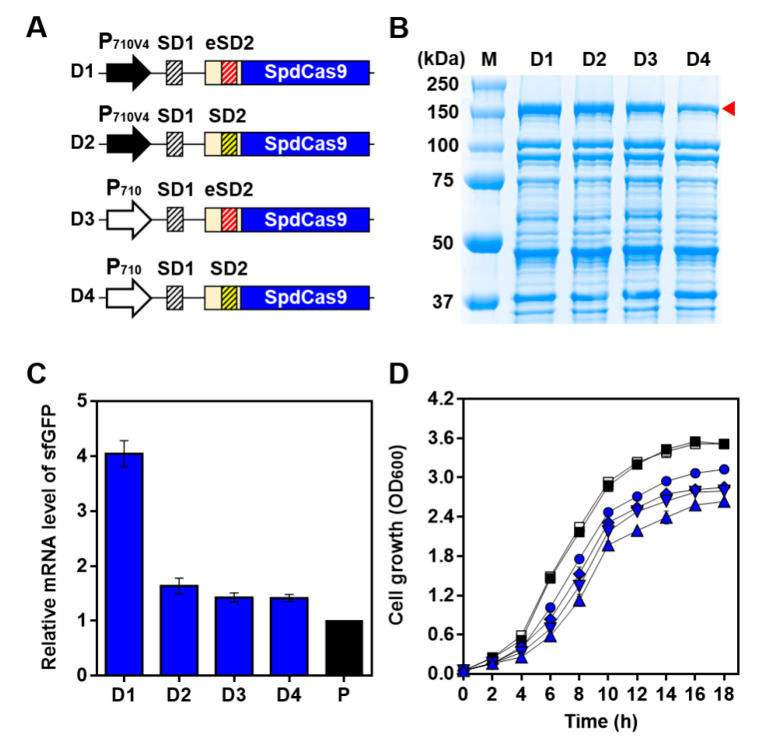
Optimization of the CRISPRi system. (**A**) Schematic illustration of the BCD versions of SpdCas9. (**B**) Analysis of SpdCas9 in the BCD system by SDS-PAGE. Lane M; molecular weight markers (kDa). Lanes D1–D4 represent *L. citreum* JW001 harboring pD1–Em, pD2–Em, pD3–Em, or pD4–Em, respectively. The arrowhead (red triangle) indicates the band of SpdCas9 (~158 kDa). (**C**) Quantification of sfGFP mRNA by qRT-PCR. P; *L. citreum* JW001. D1–D4, same as (**B**). (**D**) Time profiles of cell growth. Symbols: □; *L. citreum* CB2567 harboring pCB4270, ■; *L. citreum* JW001 harboring pCB4270–Em, ▲; *L. citreum* CB2567 harboring pD1, ▼; *L. citreum* CB2567 harboring pD2, ◆; *L. citreum* CB2567 harboring pD3, ●; *L. citreum* CB2567 harboring pD4. The growth curve was presented as an average value from the biological replicates’ cell culture.

To control SpdCas9 expression in a gradient manner, we constructed four sets of BCD combinations (D1, D2, D3, and D4) that enable the expression of SpdCas9 (without the co-expression of sgRNA) at various intensities using two different strength promoters (strong P_710V4_ and weak P_710_) and SD parts (strong eSD2 and weak SD2): D1 was constructed with P_710V4_ and eSD2; D2 with P_710V4_ and SD2; D3 with P_710_ and eSD2; and D4 with P_710_ and SD2 (Figure 3A). After the cultivation of the cells in each combination, the SpdCas9 expression level in each cell group was determined by SDS-PAGE, and we found that the expression of SpdCas9 could be fine tuned by a combination of synthetic parts in BCD platforms. D1 expressed the highest expression of SpdCas9, and a moderate expression was observed in D2 and D3; above all, the combination of weak P_710_ and weak SD2 (D4) exhibited the weakest expression of SpdCas9 (Figure 3B).

We compared the transcription level of the sfGFP gene under each combination by qRT-PCR. The comparative analysis of the mRNA clearly indicated that the combination of weak expression parts was highly effective in the down regulation of sfGFP transcription (Figure 3C). Compared with the D1 combination, exhibiting the highest SpdCas9 expression, all the other combinations (D2, D3, and D4) showed remarkably reduced levels of sfGFPs, which were similar to those of the positive control (*L. citreum* JW001) (Figure 3C). Among the four combinations, the D4 combination with a weak promoter and a weak SD showed a higher reduction (65%) in the transcription level than D1, but displayed a similar level to the positive control (Figure 3C). With these four combinations, we also confirmed that the cell growth gradually recovered as the expression of SpdCas9 decreased. Similar to the qRT-PCR results, the D4 combination showed the highest cell growth rate and recovery of cell growth, with up to 86% for the negative (*L. citreum* CB2567 harboring pCB4270) and positive controls (*L. citreum* JW001 harboring pCB4270–Em) when the stationary phase was reached (18 h) (Figure 3D).

**Figure 4:**
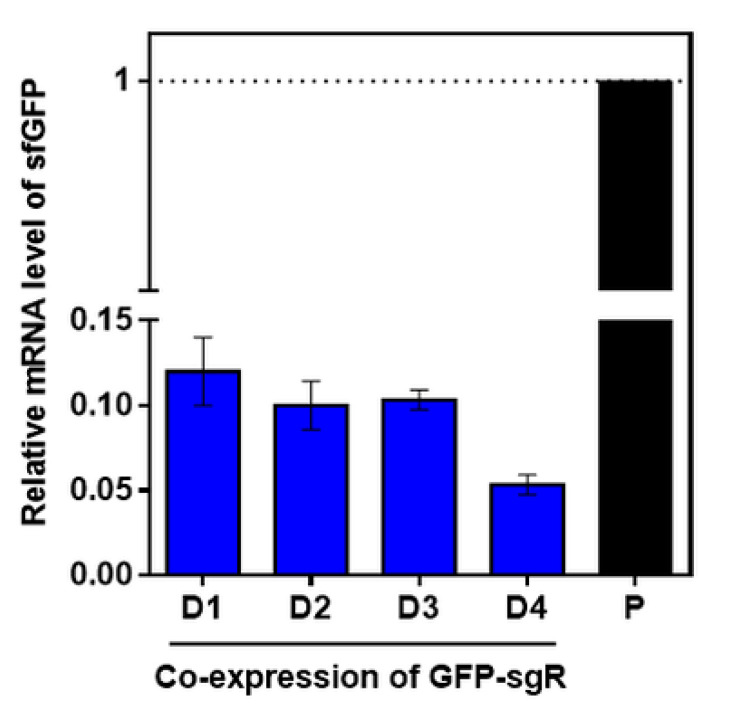
Quantification of sfGFP mRNA by qRT-PCR. P, *L. citreum* JW001; D1, *L. citreum* JW001 harboring pGFP–sgR–D1–Em; D2, *L. citreum* JW001 harboring pGFP–sgR–D2–Em; D3, *L. citreum* JW001 harboring pGFP–sgR–D3–Em; D4, *L. citreum* JW001 harboring pGFP–sgR–D4–Em. All error bars represent the standard deviation which was calculated from three repeated experiments.

We examined whether the gene knock-down efficiency of the target gene could be properly maintained in cells by cooperating with sgRNA even when the expression of SpdCas9 was reduced. Maintaining a proper concentration between sgRNA and SpdCas9 is important for the effective repression of target genes in the CRISPRi system [30]. Therefore, when GFP-sgRNA was transcribed using the strong promoter (P_710V4_), the downregulation of the sfGFP gene in each SpdCas9 expression set (D1–D4) occurred, which was verified by qRT-PCR. The comparative analysis of the mRNA revealed that the transcription level of sfGFP was remarkably reduced by the co-expression of GFP-sgRNA and SpdCas9, and its level was gradually regulated by the SpdCas9 expression level (Figure 4). Cells harboring pGFP–sgR–D1–Em (strong expression of SpdCas9) showed the highest transcription level, but the cells harboring pGFP–sgR–D4–Em (weak expression of SpdCas9) showed the lowest transcription level, which was below 5% of the positive control (*L. citreum* JW001) (Figure 4). We found that the combination of sgRNA expression under strong P_710V4_ with D4 (weak expression of SpdCas9) was the most optimized system of CRISPRi for *L. citreum*.

### 2.3. Increase in Riboflavin Production via Optimized CRISPRi System for L. citreum

**Figure 5:**
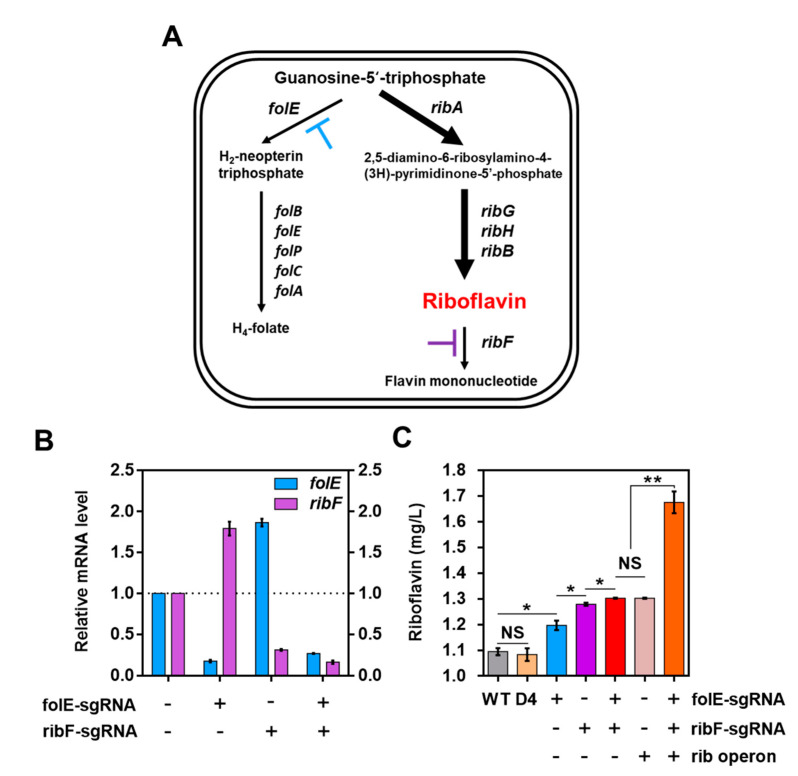
(**A**) Schematic diagram of the biosynthesis pathway of riboflavin in *L. citreum*. Blue and purple T-shaped bars represent the repression by CRISPRi with *folE* and *ribF*-specific sgRNA, respectively. (**B**) Transcription analysis of the *folE* and *ribF* genes by qRT-PCR. Blue and purple bars represent the transcription of *folE* and *ribF* genes, respectively. All the error bars represent the value of the standard deviation, which was calculated from three repeated experiments. (**C**) Identification of riboflavin accumulation via the designed CRISPRi system and the verification of the synergetic effect of riboflavin accumulation via combination with the CRISPRi system and the *rib* operon expression cassette. Wild-type (WT), *L. citreum* CB2567; D4, *L. citreum* CB2567 harboring plasmid pD4. The error bars represent the standard deviation of the samples. NS represents nonsignificant (*p*-value > 0.05), * *p*-value < 0.05, ** *p*-value < 0.01 based on the Student *t*-test.

To demonstrate the feasibility of the optimized CRISPRi system in *L. citreum*, we engineered the riboflavin biosynthesis pathway toward the enhanced production of riboflavin. Riboflavin, also known as vitamin B2, is an essential component of cellular metabolism required by humans [31]. Recently, the use of LAB as a microbial cell factory for producing riboflavin has been proposed to obtain fermented bio-enriched food [32,33,34] as the addition of the riboflavin-producing LAB to fermented foods is both feasible and economically viable in the dairy industry [35]. In the riboflavin biosynthesis pathway, guanosine-5’-triphosphate (GTP) is converted to 2,5-diamino-6-ribosylamino-4-(3H)-pyrimidinone-5’-phosphate by GTP cyclohydrolase II and is subsequently transferred to the flavin mononucleotide (FMN) synthesis pathway [36] (Figure 5A). However, GTP, the main precursor, can be also converted to H_2_-neopterin triphosphate by GTP cyclohydrolase I, and then transferred to the folate synthesis pathway (Figure 5A). Therefore, to enhance the biosynthesis of riboflavin, it is necessary to reduce the expression of the *folE* gene encoding GTP cyclohydrolase I, which can minimize the flux of GTP toward the folate synthesis pathway. In addition, a *ribF* gene, which encodes the riboflavin kinase responsible for the bioconversion of riboflavin to FMN, was chosen as the second target for downregulation, in order to reduce the loss of riboflavin due to its conversion into FMN (Figure 5A). We designed sgRNAs targeting the *folE* and *ribF* genes, which comprised 20-base sequences, complementary to the non-template target regions next to PAM sequences located at the most upstream position in each coding sequence (Table S3 and Figure S1B and S1C).

For the knock-down of both *folE* and *ribF* genes expressions by the CRISPRi, pFRdual–D4 was constructed, in which each gene-specific sgRNA was co-expressed together with the SpdCas9 (D4 platform). As the controls, two plasmids, pFolE–sgR–D4 and pRibF–sgR–D4, were also constructed for the transcription of either *folE-* or *ribF*-specific sgRNA, respectively. After the cultivation of each strain, the repression level of each gene was determined by qRT-PCR. As shown in Figure 5B, cells harboring either pFolE–sgR–D4 or pRibF–sgR–D4 showed a reduced transcription level only in the target genes (5.6-fold decrease in *folE* and 3.3-fold decrease in rib*F*), which were orthogonal to each target without any interference (Figure 5B). Moreover, the cells harboring the FRdual–D4 exhibited a transcriptional repression activity with a 3.9-fold decrease in *folE* and 6.3-fold decrease in *ribF* compared with those in WT (Figure 5B).

We examined whether the repression of *folE* and *ribF* expression via the CRISPRi system increased the production of riboflavin in *L. citreum*. After the cultivation of the cells in a shake flask, the yield of riboflavin was analyzed by HPLC. As shown in Figure 5C, by repressing the expression of the target gene via the CRISPRi system, it was clearly observed that the changes in the amount of riboflavin production occurred in a gradient manner. The downregulation of either *folE* (pFolE–sgR–D4) or *ribF* (pRibF–sgR–D4) via the CRISPRi system increased riboflavin production by 1.13-fold (1.21 ± 0.03 mg/L) or 1.17-fold (1.28 ± 0.01 mg/L), respectively, compared with WT (1.08 ± 0.02 mg/L) or with the strain that lacked the sgRNA (D4) (1.06 ± 0.03 mg/L) (Figure 5C). Although the repression of *folE* was higher than *ribF* via the CRISPRi system (Figure 5B), relatively higher riboflavin accumulation was observed when *ribF* was repressed rather than *folE* (Figure 5C). These results indicate that direct the inhibition of conversion of riboflavin to FMN is a more effective strategy to confirm the high productivity of riboflavin. Furthermore, the cultivation of *L. citreum* CB2567 harboring pFRdual–D4 for the downregulation of both the genes exhibited the enhanced production of riboflavin (1.30 ± 0.01 mg/L), which was increased by 1.20-fold compared to WT (Figure 5C). Therefore, these results verified that the developed system effectively performs multiplex gene expression regulation in *L. citreum*.

### 2.4. Increase in Riboflavin Production by Co-Expression of the Rib Operon in L. citreum

As mentioned above, the accumulation of riboflavin could be induced in cells by regulating the flux of the riboflavin synthesis pathway via the CRISPRi system. In the riboflavin synthesis pathway, the enzymatic activities required to activate the biosynthesis of riboflavin from GTP are encoded by four genes (*ribA*, *ribG*, *ribH*, and *ribB*), which are located in an operon assembly (*rib* operon) (Figure 5A), and it was previously reported that the overexpression of *rib* operon could increase the production of riboflavin [37]. We further examined whether a combination of downregulation via the CRISPRi system and the overexpression of the *rib* operon gene could exert a synergetic effect on the production of riboflavin. The expression cassette of the *rib* operon gene was inserted into the pFRdual–D4 plasmid, yielding pFRdual–Rib–D4, and after flask cultivation, the titer of riboflavin produced was determined. First, we examined the production of riboflavin in *L. citreum* CB2567 harboring pH-rib (overexpression of the *rib* operon without the CRISPRi system), and a 1.2-fold increase in riboflavin (1.30 ± 0.01 mg/L) was observed compared to the WT (Figure 5C). *L. citreum* CB2567 harboring pFRdual–Rib–D4, in which both the repression by CRISPRi system and the overexpression of the *rib* operon were combined, and exhibited a dramatic increase in riboflavin production as high as 1.7 ± 0.06 mg/L, which was a 1.53-fold increase compared to the WT (Figure 5C).

## 3. Conclusions

In the last decade, substantial advancements in the CRISPR/Cas system have provided a very powerful tool to edit the genome and regulate the expression of endogenous genes in various microorganisms. In this study, we successfully extended the potential of the dCas9-based regulation tool to *L. citreum*, a promising probiotic. It was clearly demonstrated that the transcription of target genes was significantly reduced, and metabolic pathways for riboflavin production were successfully modulated using optimized CRISPRi. The expression levels of major components (sgRNA and dCas9) of CRISPRi could be regulated by a combination of synthetic parts (promoters and SDs) of different strengths. Through the low-level expression of dCas9 and the high-level expression of sgRNA, toxicity caused by the overexpression of dCas9 could be overcome along with the downregulation of the target gene. The regulation of the gene expression using synthetic parts also allows the fine tuning of the CRISPRi system, which makes it possible to optimize metabolic pathways towards more enhanced production of the desired biomolecules. As the power of CRISPR/Cas9 is based on its easy implementation and programmability, we believe that the CRISPRi systems employed in this study will be a useful tool for the engineering of *L. citreum* and other lactic acid bacteria, which can be employed as beneficial cell factories in various bioindustry fields.

## 4. Materials and Methods

### 4.1. Bacterial Strains and Culture Conditions

All the bacterial strains used in this study are listed in Table S1. *E. coli* XL1-blue was used as a host for the gene cloning and maintenance of plasmids. *L. citreum* CB2567 [38] was used as a major host for the development of the CRISPRi system and riboflavin production. *E. coli* was cultivated in a Luria–Bertani (LB) medium (tryptone 10 g/L, yeast extract 5 g/L, and sodium chloride 10 g/L, purchased as premixed media from BD, Franklin Lakes, NJ, USA) at 37 ℃ with shaking (200 rpm). *L. citreum* CB2567 was cultivated in *Lactobacilli* MRS (De Man, Rogosa and Sharpe) medium (proteose peptone no. 3 10 g/L, beef extract 10 g/L, yeast extract 5 g/L, dextrose 20 g/L, polysorbate 80 1 g/L, ammonium citrate 2 g/L, sodium acetate 5 g/L, magnesium sulfate 0.1 g/L, manganese sulfate 0.05 g/L, and dipotassium phosphate 2 g/L, purchased as premixed media from BD) at 30 ℃ with shaking (200 rpm). Ampicillin (Amp, 100 mg/L) was added for the selection and cultivation of *E. coli*. Chloramphenicol (Cm, 10 mg/L) and erythromycin (Em, 10 mg/L) were used for the selection and cultivation of *L. citreum*.

### 4.2. Plasmid Construction and Chromosome Integration

All the plasmids used in this study are listed in Table S1. Polymerase chain reaction (PCR) was carried out using a C1000TM Thermal Cycler (Bio-Rad, Richmond, CA, USA) and primeSTAR HS Polymerase (Takara, Shiga, Japan). All the oligonucleotides used for PCR are listed in Table S2. The erythromycin resistance gene (Emr) was amplified from pSOS95 [39] by PCR with two primers (F-Erm and R-Erm). The PCR product was digested with two restriction enzymes (*Stu*I and *Xba*I), and cloned into pCB4270 and pCB4270V4, yielding pCB4270–Em and pCB4270V4–Em, respectively. For the expression of the SpdCas9 gene under the strong control ofeSD2, the eSD2–dCas9 gene was amplified from pdCas9 by PCR using three primers (F1–eSD2dCas9, F2–eSD2dCas9, and R–Not–dCas9). The PCR product (eSD2–dCas9) was digested with two restriction enzymes (*Xho*I and *Not*I) and cloned into pCB4270V4, pCB4270, pCB4270V4–Em, and pCB4270–Em, yielding plasmids pD1, pD3, pD1–Em, and pD3–Em, respectively. For the expression of the SpdCas9 gene under the control of a weak SD2, the SD2–dCas9 gene was amplified from pdCas9 by PCR with three primers (F1–SD2dCas9, F2–SD2dCas9, and R–Not–dCas9). The PCR product (SD2–dCas9) was digested with two restriction enzymes (*Xho*I and *Not*I) and cloned into pCB4270V4, pCB4270, pCB4270V4–Em, and pCB4270–Em, yielding the plasmids pD2, pD4, pD2–Em, and pD4–Em, respectively. GFP–sgRNA was amplified from pgRNA-bacteria by PCR using four primers (F1–v4sfgsgR, F2–v4sfgsgR, F3–v4sfgsgR, and R–v4sfgsgR). The PCR product was digested with the *Nar*I restriction enzyme and cloned into pCB4270–Em, pD1–Em, pD2–Em, pD3–Em, and pD4–Em, yielding plasmids pGFP–sgR–Em, pGFP–sgR–D1–Em, pGFP–sgR–D2–Em, pGFP–sgR–D3–Em, and pGFP–sgR–D4–Em, respectively. The folE–sgRNA was amplified from the plasmid pGFP–sgR–Em by PCR using four primers (F1–v4folEsgR, F2–v4folEsgR, F3–v4folEsgR, and R–v4sfgsgR). The PCR product was digested with the *Xba*I restriction enzyme, and cloned into the plasmid pD4, yielding pFolE–sgR–D4. A ribF–sgRNA was amplified from the plasmid pGFP–sgR–Em by PCR with three primers (F1–v4ribFsgR, F2–v4ribFsgR, and R–v4sfgsgR). The PCR product was digested with the *Nar*I restriction enzyme, and cloned into the plasmid pD4, yielding pRibF–sgR–D4. Plasmid pFRdual-D4 was constructed by inserting the *Nar*I-digested PCR product of ribF–sgRNA into the plasmid pFolE–sgR–D4. A *rib* operon was amplified from the genomic DNA of *L. citreum* CB2567 by PCR using four primers (F1–v4–ribD, F2–v4–ribD, F3–v4–ribD, and R–ribH). The PCR product was digested with the *Pst*I restriction enzyme, and cloned into the plasmids pCB4270 and pFRdual–D4, yielding pH–rib and pFRdual–Rib–D4, respectively.

A sfGFP gene was integrated into the chromosomal DNA of *L. citreum* CB2567 via the homologous recombination using a suicide vector pCB4270–InsfGFP. To construct pCB4270–InsfGFP, a 0.8 kb upstream region of *ldhD* gene was amplified from the chromosomal DNA of *L. citreum* CB2567 by PCR using F–LDHU and R–LDHU primers. The PCR product was digested with *Bam*HI and then ligated into pCB4270–sfGFP yielding pHldhU–sfGFP. The *ldh* gene was amplified from the chromosomal DNA of *L. citreum* CB2567 by PCR using F–LDHD and R–LDHD primers. After digestion with *Pst*I, the PCR product was cloned into pHldhU–sfGFP, yielding pH–InsfGFP. After electroporation, the integrated clones were screened on agar plates containing chloramphenicol. The generation of integrated clones was confirmed by PCR using IF–stCAT and OR–ldh primers. The correct clone was named *L. citreum* JW001.

### 4.3. Fluorescence-Activated Cell-Sorting (FACS) Analysis

After the overnight cultivation of the recombinant *L. citreum*, the cells were transferred to 5 mL of fresh MRS medium in a 50 mL tube at 1:50 dilution and grown at 30 ℃ with shaking (200 rpm). After cultivation for 12 h, the cells were harvested by centrifugation at 13,000 rpm for 5 min at 4 ℃ and washed twice with phosphate-buffered saline (PBS, containing 135 mM NaCl, 2.7 mM KCl, 4.3 mM ${\mathrm{Na}}_{2}{\mathrm{HPO}}_{4}$, pH 7.2). After suspension in the same buffer, the cells were identified using a high-speed flow cytometer (MoFlo XDP, Beckman Coutler, Miami, FL, USA). For the FACS analysis, the cells were analyzed on the basis of high fluorescence intensity detected through a 530/40 band-pass filter for the GFP emission spectrum.

### 4.4. Protein Preparation and Analysis

After culturing at 30 ℃ for 12 h in a shake flask, the cells were harvested by centrifugation (13,000 rpm, 5 min, 4 ℃) and washed with PBS. Later, the cells were disrupted by sonication (7 min with a 5 s pulse and 3 s cooling time, 20% amplitude). After harvesting the total fractions, insoluble pellets were eliminated by centrifugation (13,000 rpm, 5 min, 4 ℃) and the soluble fractions were collected from the supernatants. The protein samples were analyzed by 10% sodium dodecyl sulfate-polyacrylamide gel electrophoresis (SDS-PAGE).

### 4.5. Quantitative Reverse Transcription PCR (qRT-PCR)

mRNA transcription levels were quantified by qRT-PCR. *L. citreum* harboring each plasmid was cultivated in MRS medium for 12 h, and then the cells were harvested by centrifugation (13,000 rpm, 5 min, 4 ℃) and washed with PBS. Total RNA was extracted from the cells using the Qiagen RNeasy Mini Kit (Qiagen, Valencia, CA, USA), and the purified RNA was stored at –80 ℃ for further use. The primers sets for qRT-PCR were designed using Primer3web (http://primer3.ut.ee); the resulting primer sets are listed in Table S2. One-step qRT-PCR was performed using the One Step SYBR PrimeScript RT-PCR Kit (Takara, Japan) following the manufacturer’s instructions. qRT-PCR was carried out on a CFX-96 real-time PCR system (Bio-Rad, CA, USA) and the Cq values were determined by CFX Manager Software (Bio-Rad, CA, USA)).

### 4.6. High-Performance Liquid Chromatography (HPLC) Analysis

To measure the amount of riboflavin in the medium, the cells were pelleted by centrifugation at 13,000 rpm for 5 min at 4 °C, and the supernatant thus obtained was filtered using a 0.22 μm syringe filter (Futecs, Daejeon, Korea). The filtrated supernatant was diluted 1:10 in distilled water and analyzed by HPLC. The HPLC system (Shimadzu, Kyoto, Japan) consisted of a pump (LC-20AD), autosampler (SIL-30AC), column oven (CTO-20A), and a refractive index detector (RID-10A). A Zorbax 300SB-C18 column (150 × 4.6 mm; Agilent Technologies, PA, CA, USA), and was eluted with methanol:water (75:25 *v*/*v*), used as the mobile phase at a flow rate of 0.5 mL/min, operating at 65 °C. The chromatographic software Lab Solution (Shimadzu, Kyoto, Japan) was used for the sample acquisition and data analysis.

## Supplementary Materials

Supplementary Materials can be found at https://www.mdpi.com/1422-0067/21/16/5614/s1.

## Abbreviations

LAB	lactic acid bacteria
CRISPRi	CRISPR interference
sgRNA	single guide RNA
SpdCas9	catalytically deactivated Cas9 of *Streptococcus pyogenes*
BCD	bicistronic design
RBS	ribosome binding sites
sfGFP	superfolder green fluorescent protein
PAM	protospacer adjacent motif
qRT-PCR	quantitative reverse transcription PCR
HPLC	high performance liquid chromatography
PBS	phosphate-buffered saline
TBS-T	Tris-buffered saline with Tween-20 solution
