# Supplementary material for: Development of CRISPR Interference (CRISPRi) Platform for Metabolic Engineering of Leuconostoc citreum and Its Application for Engineering Riboflavin Biosynthesis
Source: Int J Mol Sci. 2020 Aug 5;21(16):5614. doi: 10.3390/ijms21165614 (PMC7460652; doi:10.3390/ijms21165614)
Supplement: Supplementary file 1 [file ijms-21-05614-s001.pdf]

Supplementary Information

Development of CRISPR Interference (CRISPRi) Platform for Metabolic Engineering of *Leuconostoc citreum* and Its Application for Engineering Riboflavin Biosynthesis

Jaewoo Son [1], Seung Hoon Jang [1], Ji Won Cha [1] and Ki Jun Jeong [1,2*]

[1] Department of Chemical and Biomolecular Engineering, BK21 Plus program, KAIST, 291 Daehak-ro, Yuseong-gu, Daejeon 34141, Korea; jwson90@kaist.ac.kr (J.S.); s.h.jang0314@gmail.com (S.H.J.); jiwoncha@kaist.ac.kr (J.W.C.)

[2] Institute for The BioCentury, KAIST, 291 Daehak-ro, Yuseong-gu, Daejeon 34141, Korea

* Correspondence: kjjeong@kaist.ac.kr; Tel.: +82-42-350-3934

Table 1. Bacterial strains and plasmids used in this study.

Plasmid	Description	Reference
**Strains**		
XL 1-Blue	*recA1 endA1 gyrA96 thi-1 hsdR17 supE44 relA1 lac [F´ proAB lacIqZDM15 Tn10 (Tetr)]*	Stratagene[a]
L. citreum CB2567	Wild type	[38]
JW001	*L. citreum* CB2567, Δ*ldh*D::P_{710}-sfGFP-Cm^R	This study
**Plasmids**		
pCB4270	*L. citreum-E. coli* shuttle vector, P_{710}, Amp^R, Cm^R	[19]
pCB4270-Em	*L. citreum-E. coli* shuttle vector, P_{710}, Amp^R, Em^R	This study
pCB4270-sfGFP	Monocistronic, P_{710}, sfGFP, Amp^R, Cm^R	[19]
pCB4270V4	*L. citreum-E. coli* shuttle vector, P_{710V4}, Amp^R, Cm^R	This study
pCB4270V4-Em	*L. citreum-E. coli* shuttle vector, P_{710V4}, Amp^R, Em^R	This study
pdCas9	Plasmid carrying SpdCas9 catalytic site mutant	Addgene, USA
pgRNA-bacteria	Plasmid carrying customized tracrRNA for bacterial gene knock-down	Addgene, USA
pSOS95	Acetone operon, *repL* gene, ColE1 origin, Amp^R, MLS^R, Em^R	[39]
pHldhU-sfGFP	pCB4270-sfGFP derivative containing 0.8 kb homologous region *Bam*HI upstream fragment of *ldhD*	This study
pH-InsfGFP	pCB4270ldhU-sfGFP derivative suicide vector containing 0.8 kb homologous region *Bam*HI upstream fragment of *ldhD* and 1 kb homologous region *Pst*I internal fragment of *ldhD*, Amp^R, Cm^R	This study
pGFP-sgR-Em	pCB4270-Em derivative carrying P_{710V4}, sfGFP targeting sgRNA, Amp^R, Em^R	This study
pD1-Em	pCB4270V4-Em derivative carrying bicistronic, P_{710V4}, eSD2, SpdCas9, His tag, Amp^R, Em^R	This study
pD2-Em	pCB4270V4-Em derivative carrying bicistronic, P_{710V4}, SD2, SpdCas9, His tag, Amp^R, Em^R	This study
pD3-Em	pCB4270-Em derivative carrying bicistronic, P_{710}, eSD2, SpdCas9, His tag, Amp^R, Em^R	This study
pD4-Em	pCB4270-Em derivative carrying bicistronic, P_{710}, SD2, SpdCas9, His tag, Amp^R, Em^R	This study
pD1	pCB4270V4 derivative carrying bicistronic, P_{710V4}, eSD2, SpdCas9, His tag, Amp^R, Cm^R	This study
pD2	pCB4270V4 derivative carrying bicistronic, P_{710V4}, SD2, SpdCas9, His tag, Amp^R, Cm^R	This study
pD3	pCB4270 derivative carrying bicistronic, P_{710}, eSD2, SpdCas9, His tag, Amp^R, Cm^R	This study
pD4	pCB4270 derivative carrying bicistronic, P_{710}, SD2, SpdCas9, His tag, Amp^R, Cm^R	This study
pGFP-sgR-D1-Em	pD1-Em derivative carrying bicistronic, P_{710V4}, eSD2, SpdCas9, His tag, P_{710V4}, sfGFP targeting sgRNA, Amp^R, Em^R	This study
pGFP-sgR-D2-Em	pD2-Em derivative carrying bicistronic, P_{710V4}, SD2, SpdCas9, His tag, P_{710V4}, sfGFP targeting sgRNA, Amp^R, Em^R	This study

pGFP-sgR-D3-Em	pD3-Em derivative carrying bicistronic, P_{710}, eSD2, SpdCas9, His tag, P_{710V4}, sfGFP targeting sgRNA, Amp^R, Em^R	This study
pGFP-sgR-D4-Em	pD4-Em derivative carrying bicistronic, P_{710}, SD2, SpdCas9, His tag, P_{710V4}, sfGFP targeting sgRNA, Amp^R, Em^R	This study
pFolE-sgR-D4	pD4 derivative carrying bicistronic, P_{710}, SD2, SpdCas9, His tag, P_{710V4},*folE* targeting sgRNA, Amp^R, Cm^R	This study
pRibF-sgR-D4	pD4 derivative carrying bicistronic, P_{710}, SD2, SpdCas9, His tag, P_{710V4}, *ribF* targeting sgRNA, Amp^R, Cm^R	This study
pFRdual-D4	pFolE-sgR-D4 derivative carrying bicistronic, P_{710}, SD2, SpdCas9, His tag, P_{710V4},*folE* targeting sgRNA, P_{710V4}, *rib*F targeting sgRNA, Amp^R, Cm^R	This study
pH-rib	pCB4270 derivative carrying monocistronic, P_{710V4}, *rib* operon, Amp^R, Cm^R	This study
pFRdual-Rib-D4	pFRdual-D4 derivative carrying bicistronic, P_{710}, SD2, SpdCas9, His tag, P_{710V4},*fol*E targeting sgRNA, P_{710V4}, *rib*F targeting sgRNA, monocistronic, P_{710V4}, *rib* operon, Amp^R, Cm^R	This study

Table S2: Oligonucleotides primers used in this study

Primer	Sequence (5' to 3')
F-LDHU	GGATCCAATGTTATGTTGCGAATATATTTTAAATAA
R-LDHU	GGATCCAAGATCCTCCAAAATTTATATTACCA
F-LDHD	CTGCAGGACTTATTGACTAAGCTCGGG
R-LDHD	CTGCAGTTTCATGGCACCAAGTGC
IF-stCAT	GGACTTCATTTACTGGGTTT
OR-ldh	ATATTGTGCAATGAAACCAGC
F1-v4sfgsgR	TGAGTATAACCAGAACATCTAATTCAACAAGAATTGTTTTAGAGCTAGAAATAGCAAGTTAAAATAAGG
F2-v4sfgsgR	AAGTTAATAGGTGTTTTTAGCCTGAAGTGTTATAATGAGTATAACCAGAACATCTAATTCAACAA
F3-v4sfgsgR	CCGGCGCCGAGAACAGTTTAAAGTTAATAGGTGTTTTTAGCCTGAAGT
R-v4sfgsgR	GGCGCCAGTTCACCGACAAACAACAGAT
F1-v4folEsgR	CAGAAATATTTGACGCCCTGCACGAGTTTTAGAGCTAGAAATAGCAAGTTAAAATAAG
F2-v4folEsgR	AGTTAATAGGTGTTTTTAGCCTGAAGTGTTATAATGAGTATAACCAGAAATATTTGACGCCCTGC
F3-v4folEsgR	AATCTAGAGAGAACAGTTTAAAGTTAATAGGTGTTTTTAGCCTGAAGT
F1-v4ribsgR	AATGAGTATAACCAGAAGGCTGTTTGAAATTTTGAATGTTTTAGAGCTAGAAATAGCAAGTTAAAATAAG
F2-v4ribsgR	GAGAACAGTTTAAAGTTAATAGGTGTTTTTAGCCTGAAGTGTTATAATGAGTATAACCAGAAGGCTGTTTG
F1-eSD2dCas9	ATCTTAATCATGGAAGGGAGGGTTTTTAATGGATAAGAAATACTCAATAGGCTTAGCTA
F2-eSD2dCas9	GGCTCGAGATGAAAGCAATTTTCGTACTGAAACATCTTAATCATGGAAGGGAGGGT
F1-SD2dCas9	ACATCTTAATCATGCAAAGGAGGTGTTTTAATGGATAAGAAATACTCAATAGGCTTAGC
F2-SD2dCas9	AGCTCGAGATGAAAGCAATTTTCGTACTGAAACATCTTAATCATGCAAAGGAGGTG
R-Not-dCas9	GCGCGGCCGCTTATTAGTGG
F-RT-sfGFP	TGCTACAAACGGAAAACTCA
R-RT-sfGFP	GCGTTCCTGTACATAACCTT
F-RT-folE	ACCTCGCTTGGTTGATTGGG
R-RT-folE	CGTGTTTGTTTGCGTCCCTG
F-RT-ribF	TCCACATCCAAGTGTCGCAT
R-RT-ribF	CCTGTGGTGATAAACCCGCT
F-RT-16srRNA	TATGTCCCGAGCGTTATCCG
R-RT-16srRNA	TCTACGCATTCCACCGCTAC

F-Erm	AATCTAGAGCATTGACGTCAGATACTGCA
R-Erm	TTAGGCCTCTGCAGGAGCTTTGGCTAA
F1-v4-ribD	ATAATGAGTATAACCAGAAGAAAGGATAGAAAAAATGGATGATTTAACATATATGGCATTAGCA
F2-v4-ribD	GTGTTTTTAGCCTGAAGTGTTATAATGAGTATAACCAGAAGAAAGGATAGAAA
F3-v4-ribD	GACTGCAGGAGAACAGTTTAAAGTTAATAGGTGTTTTTAGCCTGAAGTGTTATAATGA
R-ribH	TTCTGCAGTCAGTGGTGGTGGTGGTGGTGAGACAGTTGTTTTATGTCATCATATAAACTT

Table S3. sgRNA used in this study

sgRNA name	Target location	Spacer sequence (5’ to 3’)	PAM sequence (5’ to 3’)
GFP-sgRNA	CDS of sfGFP	AATTCTTGTTGAATTAGATG	CCC
folE-sgRNA	CDS of *folE*	ATATTTGACGCCCTGCACGA	CCC
ribF-sgRNA	CDS of *ribF*	GGCTGTTTGAAATTTTGAAT	CCA

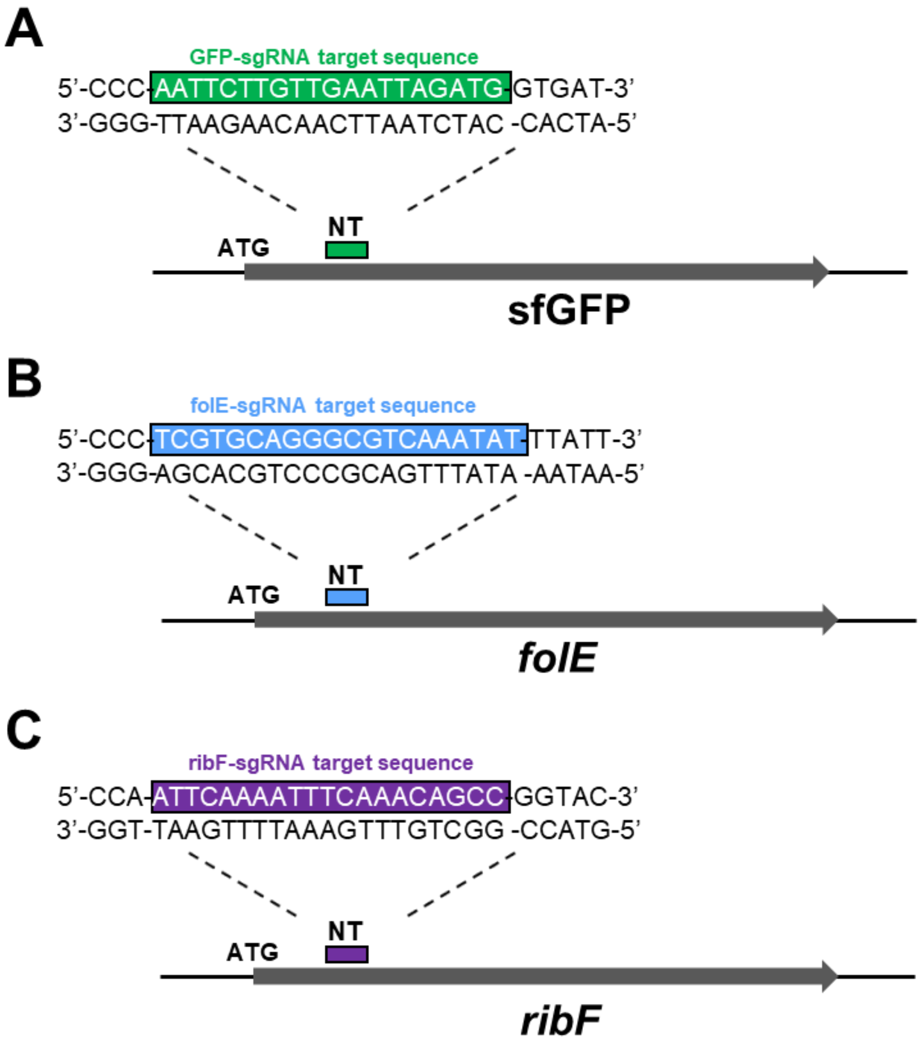

Figure S1. Schematic of synthetic guide RNAs target sequence used in this study. NT represents non-template strand of target gene. **(A)** Target sequence of GFP-sgRNA for sfGFP gene. **(B)** Target sequence of folE-sgRNA for *folE* gene. **(C)** Target sequence of ribF-sgRNA for *ribF* gene.
